# Rapid and sensitive detection of *Pseudomonas aeruginosa* by isothermal amplification combined with Cas12a-mediated detection

**DOI:** 10.1038/s41598-023-45766-0

**Published:** 2023-11-06

**Authors:** Siyi Huang, Xianfeng Wang, Xinchong Chen, Xiaoyu Liu, Qiuqing Xu, Lijun Zhang, Guangtao Huang, Jun Wu

**Affiliations:** 1 Department of Burn and Plastic Surgery, Shenzhen Institute of Translational Medicine, The First Affiliated Hospital of Shenzhen University, Shenzhen, 518035 China; 2 Wuxi School of Medicine, Jiangnan University, Wuxi, 214122 China; 3 Department of Burn and Plastic Surgery, The Affiliated Hospital of Zunyi Medical University, Zunyi, 563000 China

**Keywords:** Microbiology, Clinical microbiology

## Abstract

CRISPR based technologies have been used for fast and sensitive detection of pathogens. To test the possibility of CRISPR based detection strategy in *Pseudomonas aeruginosa* infections, a combined method of recombinase polymerase amplification followed by Cas12a-mediated detection via fluorescence reader or lateral flow biosensor (named Cas12a-RCFL) has been established in this study. The Cas12a-RCFL can detect as low as 50 CFU/mL *Pseudomonas aeruginosa.* The whole detection process can be finished within one hour with satisfied detection specificity. Cas12a-RCFL also shows good sensitivity of detecting *Pseudomonas aeruginosa inStaphylococcus aureus* and *Acinetobacter baumannii* contaminated samples. For the detection of 22 clinical samples, Cas12a-RCFL matches with PCR sequencing result exactly without DNA purification. This Cas12a-RCFL is rapid and sensitive with low cost, which shows good quality to be adopted as a point-of-care testing method.

## Introduction

*Pseudomonas aeruginosa*is a Gram-negative opportunistic human pathogen, accounting for up to 20% of infections in many hospitals^1–3^. Cystic fibrosis (CF) and immunosuppression patients are especially susceptible to *P. aeruginosa* infections. *P. aeruginosa* is also the leading cause of infection in burn patients. Except for the skin barrier being damaged, there are two additional factors contributing to the high rate of *P. aeruginosa* infections among burn patients. First, Pseudomonas level in gastrointestinal microbiome clearly increased after the burn injury^4^. Second, sera from burn patients could increase biofilm formation of *P. aeruginosa,* while inhibiting other species^5^*.*

Early diagnosis is crucial for the infections of *P. aeruginosa,* which may even reverse clinical outcomes. Conventional methods, such as bacterial culture and biochemical mediated identification require 3–5 days. Nucleotide-based detection methods could shorten the time to hours, such as polymerase chain reaction (PCR) and real-time PCR. Rapid, sensitive, and instrument-free detection strategy is needed for the early diagnosis of *P. aeruginosa* infections^6^.

Isothermal nucleic acid amplification technology such as loop-mediated isothermal amplification (LAMP) and recombinase polymerase amplification (RPA) have been developed for rapid amplification at a constant temperature^7–9^. Compared with PCR, the advantage of these two isothermal nucleic acid amplification technology is that the reaction can be achieved at constant temperature, and only a water bath pot is required to complete the reaction, rather than a precision instrument^9^. Clustered regularly interspaced short palindromic repeats (CRISPR) based technologies have been also used for fast and sensitive detection of viral infections and pathogen contamination^10^. In 2018, Doudna team reported that Cas12a can not only cut the target DNA it binds to, it can also cut other non-target single stranded DNAs. This indiscriminate cutting activity depends on the activation of Cas12a ^11^. Using single stranded DNA labeled with fluorescence group and quenching group at two ends as a reporter, Doudna team has successfully detected HPV virus with the Cas12a, achieving detection sensitivity of 92%^11^.

In this study, we developed a rapid and sensitive detection method for *P. aeruginosa*infections with the combination of isothermal nucleic acid amplification and Cas12-mediated detection. The whole detection process can be finished within 1 h and requires minimal instrument. This detection method shows an excellent potential for use as point-of-care testing.

## Method and materials

### Reagents and bacterial strains

All the DNA strands (Table S1) were synthesized by Sangon Biotech Co., Ltd (Shanghai, China). LbCas12a and RNA enzyme inhibitors were purchased from NEB UK, DNA extraction kit were from Tiangen (Tianjin, China), recombinase polymerase amplification (RPA) kit of TwistAmp ®Basic and qPCR kit were purchased from Vazyme (Nanjing, China). The structure and sequence of crRNA were designed and screened on a professional website named CHOPCHOP (http://chopchop.cbu.uib.no/). crRNA was ordered from Sangon Biotech Co., Ltd (Shanghai, China). The feasibility verification of crRNA was performed via the process that the CRISPR/Cas12a system provides a trans-cleavage for foreign ssDNA-FQ to release a fluorescence signal. The lateral flow strips integrated with colloidal gold probe, capture antibody and streptavidin were purchased from Lesunbio Co. Ltd. (Wuxi, China).All bacterial strains (listed in Table S2) were isolated and maintained in our laboratory. All isolates were verified by PCR and 16S rRNA gene sequencing.

### Bacterial culture and DNA genome extraction

Each of the bacteria strains was steaked onto LB plates from freezer stocks held at – 80°C and grown at 37 °C for 16 h to form single colonies. Single colonies were picked and grown in tubes containing 2 mL of liquid LB medium and were shaken for 16 h at 37 °C (180 rpm).Kit-based method and heat-treated method were employed to obtain bacterial genomic DNA (gDNA). As to the kit-based method, gDNA was extracted following the instruction of bacterial DNA extraction Kit (No. DP302, Tiangen, China).Bacterial cells were harvested by centrifugation (13,400*g*, 1 min) of 1 mL of cell suspension. The purified DNA was eluted twice with 50 μL deionized water. The heat-treated method was employed for crude DNA extractions from clinical samples. All swabs were first blistered into respective sterilized EP tube and gently shaken. The instrument used for heating was a metal bath, the instrument was preheated to 100 °C, then 300 μL of nucleic-free water that partially dissolved the swab was immediately inserted into metal bath. After 8 min, the lysate containing crude gDNA was collected by centrifugation at 11,000*g* for 1 min and supernatant was used as a template for RPA reaction.

### RPA assay

An RPA assay was carried out in a 10 μL reaction mixture (TwistAmp® Basic). Each reaction was carried out at 42 °C for 20 min. The reaction mixture concentrations are as follows: 29.5 μL of rehydration buffer to dissolve the lyophilized powder (containing recombinase polymerase for a 50 μL reaction), 1μL of primer mixture (0.5 μM primer-F, 0.5 μM primer-R, Table S1), 2 μL of extracted DNA template and 1 μL of MgOAC (280 mM) as an initiator.

### qPCR assay

Real-time PCR detection (Thermo Fischer, United States) system was used to detect *P. aeruginosa* in water or LB swab sample. The qPCR reaction mixtures contained 10 µL of SYBR Master Mix (Vazyme, China), 0.8 µL of each primer (5 µM), 2 µL of DNA template, and 6.4 µL of nuclease-free water. Program settings: 95 °C for 60 s, 40 cycles of 95 °C for 10 s and 60 °C for 30 s.

### Protocol of CRISPR/Cas12a detection

The detection system of CRISPR/Cas12a fluorescence system includes nucleic free water 10.5 μL, RNase inhibitor (1U/ μ L), 2 μ L 10 × NEBuffer 2.1 × 2 μ L LbCas12a (1 μ M), 2 μ L crRNA (2 μ M), 0.5 μ L fluorescence labeled ssDNA signal probe (10 μ M). Finally, 2 μ L of RPA amplification products were added and mixed evenly and centrifuged for 3 s and incubated at 37 °C in a qPCR instrument (Thermo Fischer), the HEX fluorescence channel was used to record the fluorescence signal intensity of the samples every 1 min (record 60 min continuously).

As to the lateral flow detection assay, the operating procedure was concordant with the steps mentioned above, except that the fluorescence labeled ssDNA signal probe was replaced by lateral flow ssDNA signal probe that is labeled on opposing ends with FAM and biotin. The final RPA and CRISPR/Cas12a mixture were incubated at 37 °C and then the reaction mixture was diluted 5 times with ddH_2_O. The lateral flow strips were inserted into the solution for 3 min and taken out for observation or photograph.

### Human swab detection applications

The crude DNA extractions of 22 clinical isolates of *P. aeruginosa* were tested, respectively, by Cas12a-RCFL and PCR, analyzing the detection rates of Cas12a-RCFL and qPCR. The established detection method and the qPCR method were used to test the samples separately, and the test results of the two test methods were compared.

### Statistical analysis

All bars of experimental results are shown as mean ± SD. When only two groups were compared, statistical significance was assessed using an unpaired Student’s t-test. Significance was indicated as *p* < 0.05. Statistical analyses were conducted using GraphPad Prism 5.5.

### Ethics statement

The study was approved by the Ethics Committee at Zunyi Medical University (Ethics Committee Approval No. KLLY-2021-101). Informed consent was obtained from the patients before the procedure. All the authors confirmed that all experiments were performed in accordance with relevant guidelines and regulations.

## Results

### Scheme of Cas12a-RCFL detection

**Figure 1:**
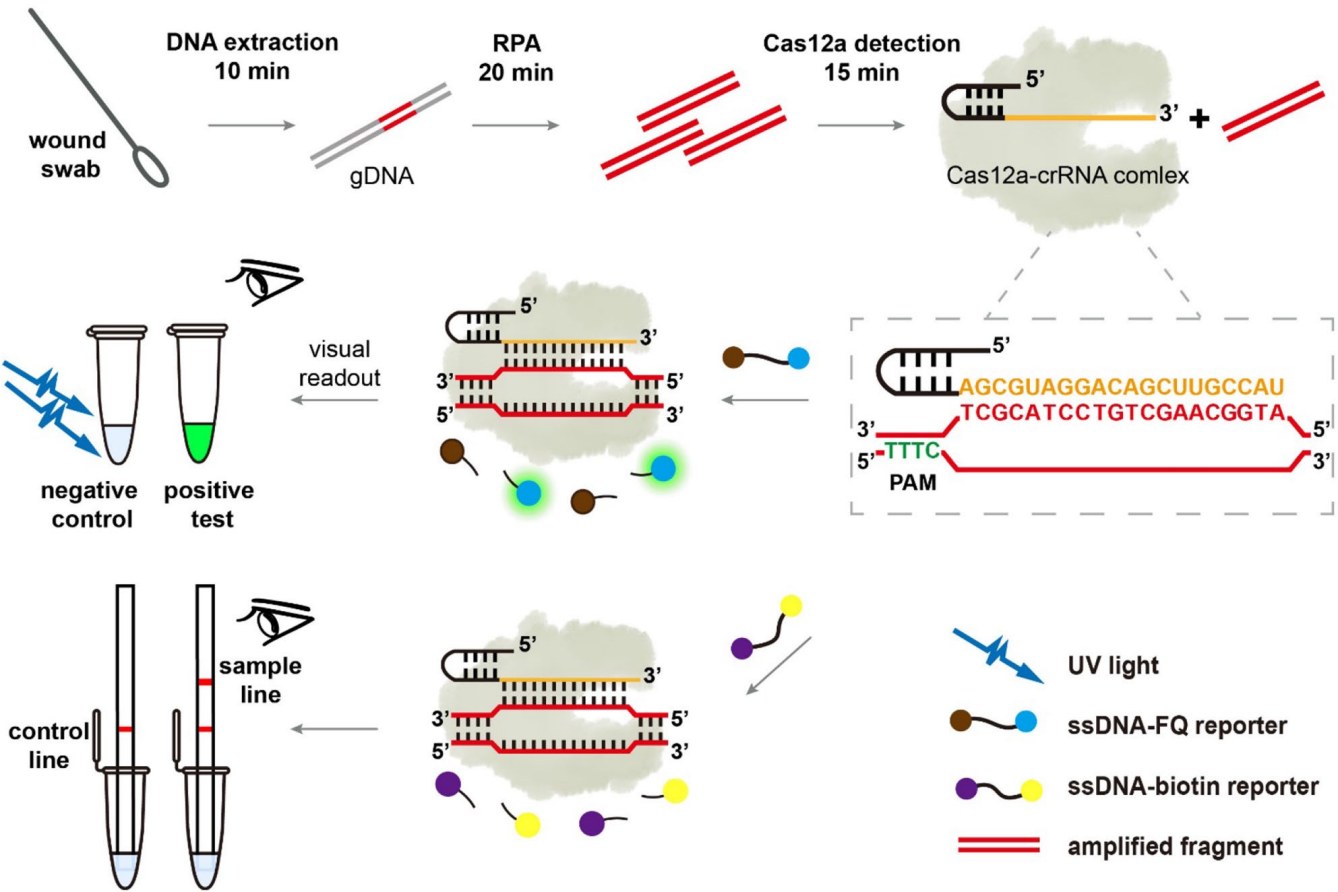
schematic illustrating DNA extraction from clinical sample to *Pseudomonas aeruginosa* identification by RPA-Cas12a assay.

Figure 1 presents the steps and principle of the RPA-assisted CRISPR/Cas12a detection strategy. First, clinical samples are enriched, and crude or purified genomic DNA (gDNA) of bacteria are extracted. Then, RPA assay is used to amplify the signal of the target gene using gDNA as template. Based on the principle of complementary base pairing, the free target DNA was then bound to the Cas12a-crRNA binary complex, activating the accessory cleavage activity of Cas12a. The Cas12a can hydrolyze the ssDNA reporter probes (labeled with 5’HEX and 3’BHQ1). The 5’HEX and the 3’BHQ1 are separated and then the fluorescence signal is generated. The relative fluorescence unit (RFU) value can be obtained in the fluorescence reading instrument. Besides, under the illumination of a portable UV lamp, the fluorescent emission of the reaction tube can be seen by the naked eye. Our assays are also adapted for non-UV light detection via lateral flow strip based upon degradation of an ssDNA-biotin reporter as described in Method and Materials. Therefore, this method can realize naked-eye detection of target genes through the highly selective RPA and the signal amplification by Cas12a mediated reporter molecule cleavage.

### Verification of the trans-cleavage capability of Cas12a

**Figure 2:**
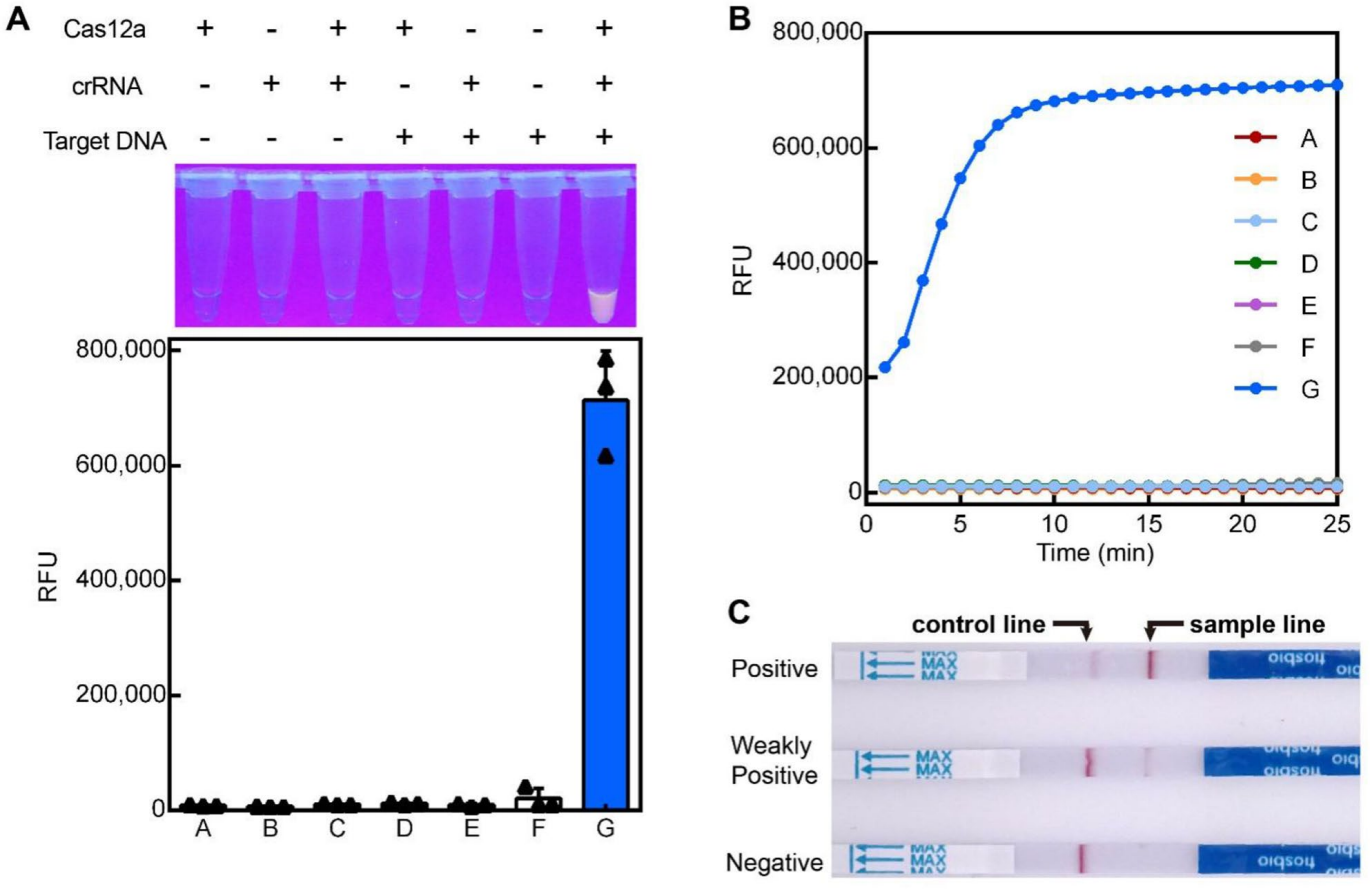
Verification of Cas12a activity for *P. aeruginosa*detection; (**A**) Fluorescence analysis of the feasibility of Cas12a for *P. aeruginosa*detection. (**B**) Real-time kinetic fluorescence analysis of target DNA induced Cas12a/crRNA cleavage. (**C**) Feasibility of RPA-assisted CRISPR/Cas12a lateral flow sensor for the detection of *P. aeruginosa.*

First, we tested whether this method could be utilized to detect *P. aeruginosa*. As the experimental results shown in Fig. 2A,B, when Cas12a and crRNA were present, presumably forming a Cas12a-crRNA complex, the Cas12a exhibited good nuclease activity upon recognition of target DNA via crRNA, as shown by the elevated fluorescent signal which is an indication of probe DNA reporter cleavage. In contrast, there was almost no fluorescent signal when Cas12a, crRNA, or target DNA absent. As shown in Fig. 2A, LbCas12a shows visible fluorescence under the guidance of crRNA in the presence of target DNA. As shown in Fig. 2C, the ssDNA probe in the Cas12a system is further replaced by a biotin-labeled strip probe, and the detection bands were present only in positive or weakly positive samples. All of these illustrates the amenability of Cas12a-RCFL strategy for the detection of *P. aeruginosa*.

### Analytical sensitivity

**Figure 3:**
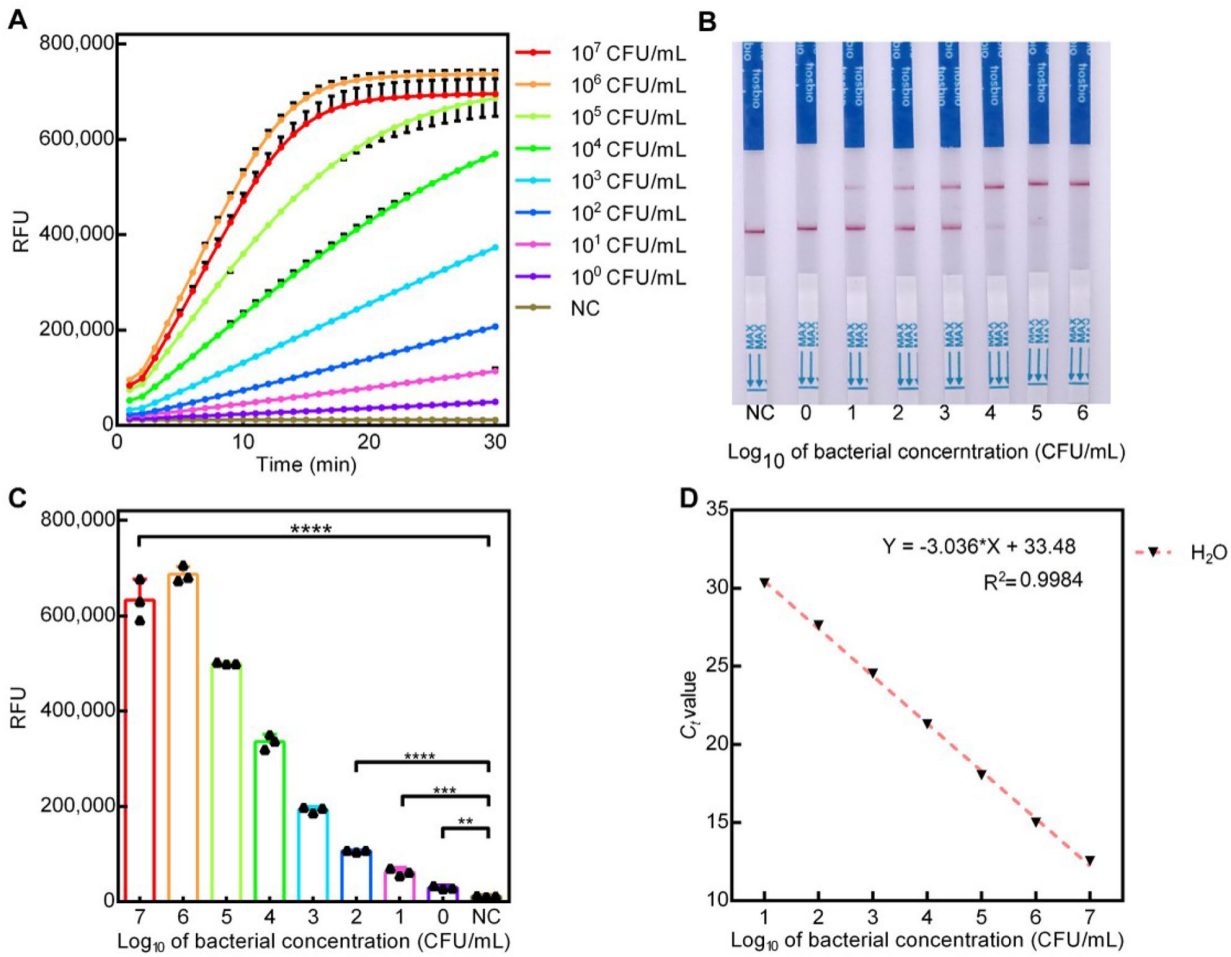
Sensitivity analysis of Cas12a- RCFL and Real-time PCR on *P. aeruginosa*. (**A**) Real-time kinetic measurement of Cas12a-RCFL assay in different concentrations of *P. aeruginosa* (5 to 5 × 10^7^ CFU/mL). (**B**) lateral flow sensor of Cas12a- RCFL assay in different concentrations of *P. aeruginosa* (5 × 10^0^ CFU/mL to 5 × 10^6^ CFU/mL). (**C**) Fluorescent intensity (n = 3) at 15th minute. (**D**) Linear analysis of *P. aeruginosa* detection by real-time PCR. n = 3 technical replicates, bars represent mean ± SD.

The sensitivity of Cas12a-RCFL was evaluated using different concentrations of *P. aeruginosa*. An example time trace with fluorescence intensity curves in the presence of varying bacterial concentrations is shown in Fig. 3A. Using the dynamic curve, we further note that differences at 15th minute were statistically significant (Fig. 3C). Subsequently, 15-min cutoff was conducted in the later RCL experiment (RCL refers to the RPA combined with Cas12a-LFA method and RCF refers to the RPA combined with Cas12a-Fluorescence method.). In the RCL experiment, the signals at the test line occurred in the 10^1^ CFU group and became stronger as increasing the bacteria concentration (Fig. 3B). The limit of detection (LOD) for the RCF and RCL was 5 CFU/mL and 50 CFU/mL, respectively. The *C*_t_ values for *P. aeruginosa* from H_2_O and corresponding linear aggression analysis are shown in Fig. 3D. Moreover, not all qPCR assays produced positive results when using templates of 10^0^ CFU/mL (1 positive results out of 3 samples). The LOD for qPCR was 50 CFU/mL. The amount of *P. aeruginosa* used in this test was quantified by plate colony counting method. Other RPA methods like RPA-LFS assay for *P. aeruginosa* detection (Yang et al., 2021) exhibits the LOD of about 3.5 CFU/reaction (about 70 CFU/mL in 50 μL RPA reaction). Therefore, the Cas12a-RCFL has a advantages in sensitivity comparing with RPA-LFS assay. Therefore, CRISPR/Cas12a is itself a signal amplification system. In this work, Cas12a-RCFL combines the CRISPR/Cas12a and RPA together, realizing the cascade signal amplification. Figure R1 reveals the sensing principle of CRISPR-based lateral flow readout. The visual probe is gold-NP-anti-FAM antibody. The total amount of gold-NP-anti-FAM antibody is constant and contains two parts that are both captured on control line and sample line. In the absence of target DNA, the CRISPR/Cas12a system can not be activated. The intact bridging reporter (5′-FAM-ssDNA-3′-Biotin) can capture all gold-NP-anti-FAM antibody on the control line by the specific binding between biotin and streptavidin, which exhibits an obvious band in control line. Contrarily, the existence of target DNA can induce mass of RPA products, which activates the trans-cleavage of CRISPR/Cas12a to digest the bridging reporter. Due to the breakage of bridging reporter, a part of gold-NP-anti-FAM antibody just binding with FAM can not be captured on control line, but captured on sample line. With the increasing concentration of target, the visual band on sample line will become more obvious, and the band on control line will fade away gradually. Therefore, the control line is invisible at high concentration of target in strips #6.

### Analytical specificity

**Figure 4:**
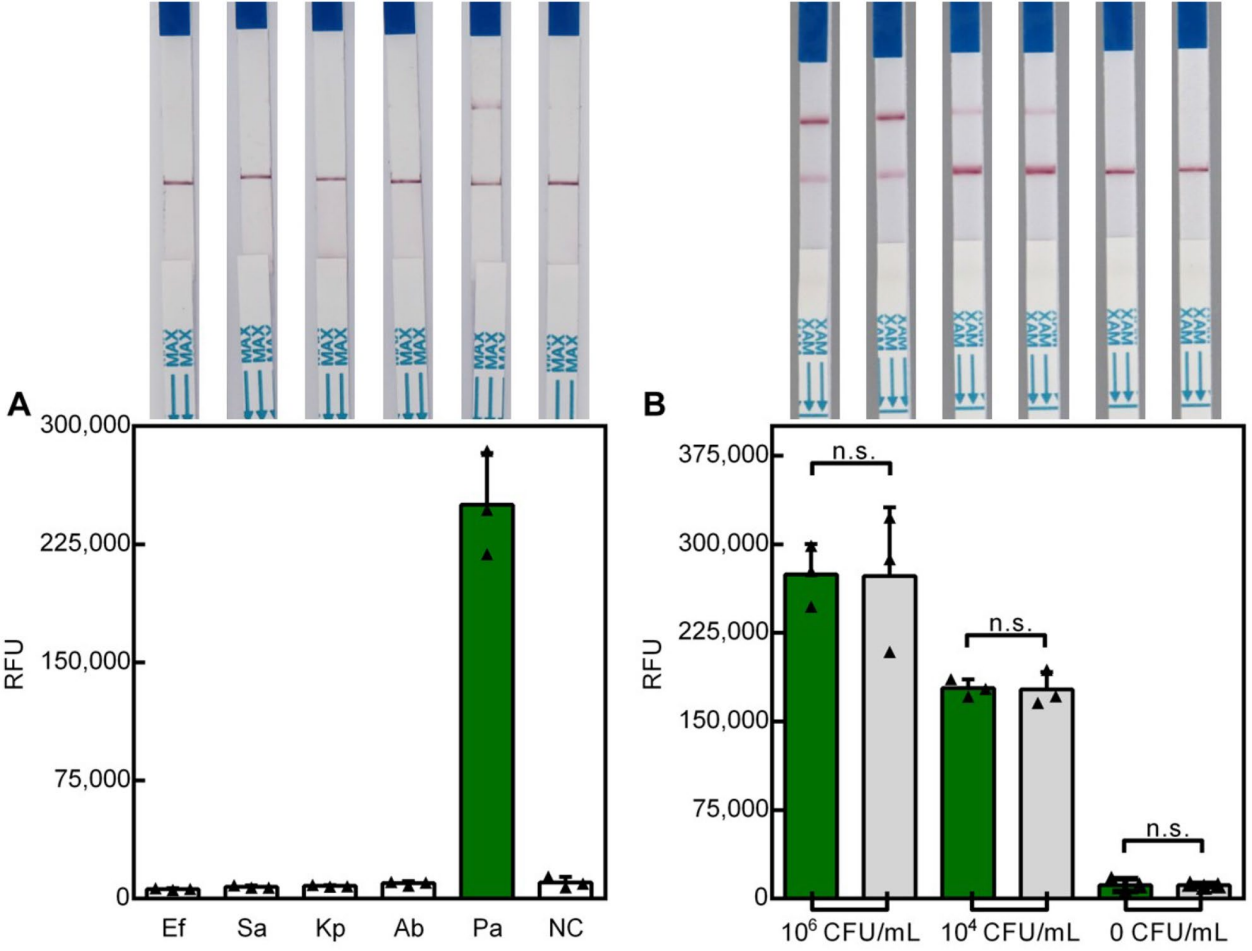
(**A**) Specificity analysis of *P. aeruginosa* in Cas12a-RCF assay among five different bacteria. End-point fluorescence and are shown here. n = 3 biological replicates; NC nontarget control; bars represent mean ± SD (**B**) Assessment of interfering from *A. baumannii* and *S. aureus*.Light gray bar represents contamination group.

The specificity of Cas12a-RCFL for *P. aeruginosa* was evaluated using gDNA from the ESKAP pathogens, including *Enterococcus faecium*, *Staphylococcus aureus*, *Klebsiella pneumoniae*, *Acinetobacter baumannii*, and *P. aeruginosa* species. As shown in the Fig. 4A, the detection system only responded to *P. aeruginosa*, and other four bacterium showed no significant changes in fluorescence intensity in the fluorescence reader or visual band in sample line. In order to simulate the situation of clinical multiple infection, *A. baumannii* and *S. aureus* at 10^6^ CFU/mL each was mixed with *P. aeruginosa* as interfering bacteria. Our detection methos show no difference between pure *P. aeruginosa* samples and contamination samples (Fig. 4B). This indicate that this method can detect *P. aeruginosa* from other common clinical pathogens, which benefits from the high selectivity of crRNA-Cas12a complex to the target DNA.

### Detection of spiked wet swabs and clinical samples

**Figure 5:**
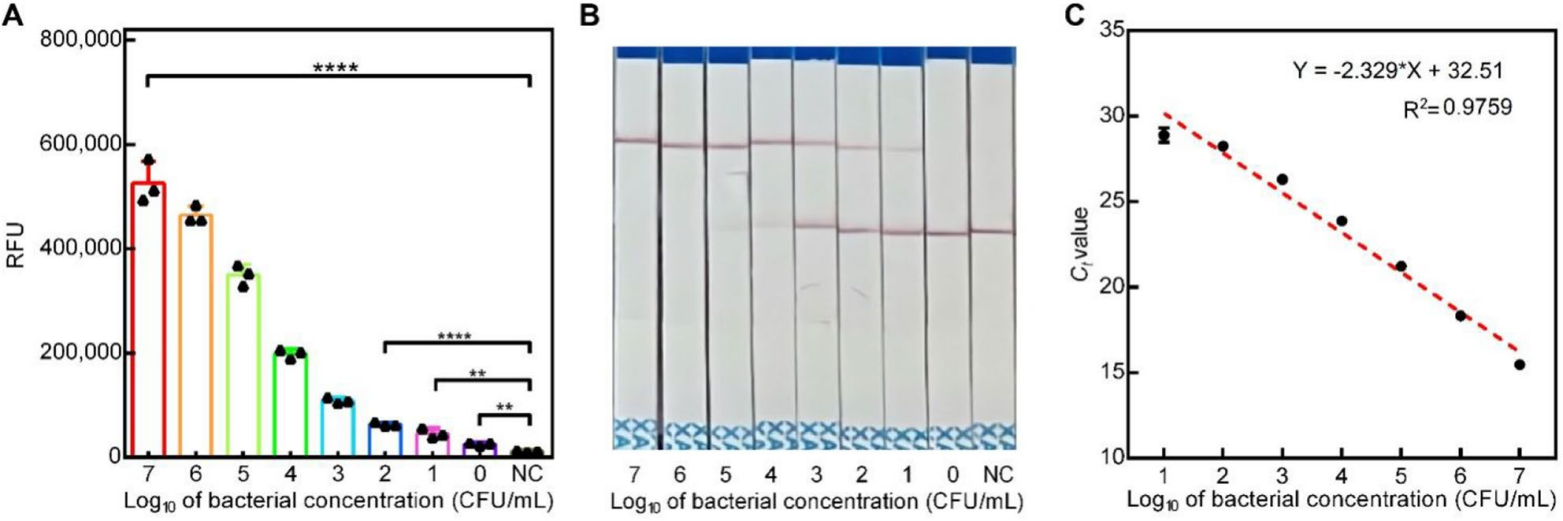
Feasibility of the Cas12a-RCF for detection of *P. aeruginosa* from LB swabs. (**A**), (**B**) Cas12a-RCFL sensitivity analysis on *P. aeruginosa* from LB swabs. (**C**) Linear analysis of *P. aeruginosa* spiked LB swabs detection by real-time PCR. n = 3 technical replicates, bars represent mean ± SD.

**Figure 6:**
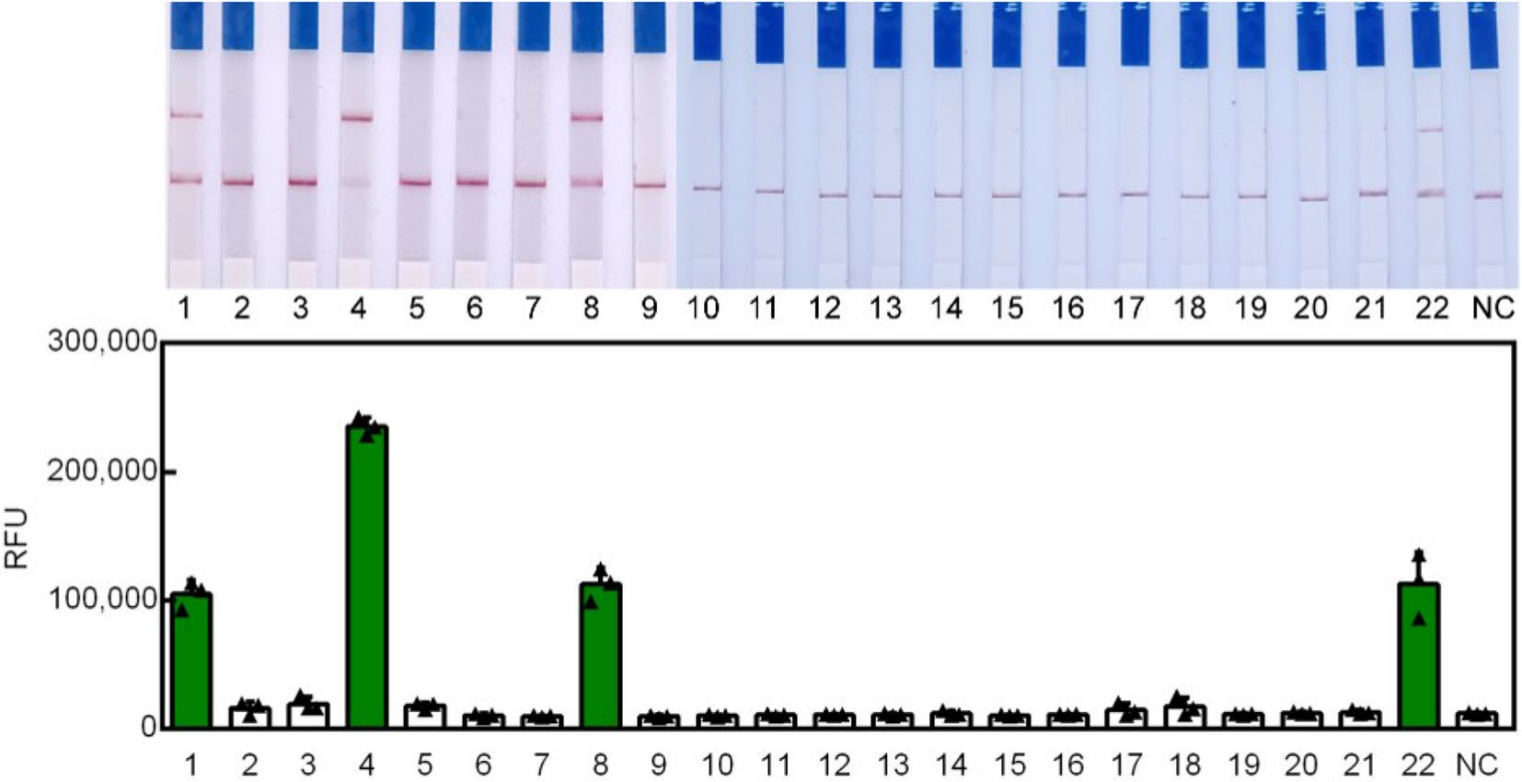
The real applications of Cas12a-RCFL for detection of *P. aeruginosa* from clinical wound samples.

As shown in the Fig. 5, clean LB was spiked with serially diluted P. aeruginosa (10^7^–10^0^ CFU/mL) and then subjected to Cas12a-RCFL reaction. Surprisingly, the LOD for Cas12a-RCFL of LB swabs gave the same results of the *P. aeruginosa* spiked H_2_O, which was 5 CFU/mL. To investigate the practicabilityof this method, wound swabs of clinical samples were collected and tested using the Cas12a-RCFL method. The total number of clinical samples is 22, which includes 4 positive samples and 18 negative samples. It further improves the accuracy of detection. The fluorescent and visual results were displayed in the Fig. 6, which was coincident with the results of PCR method. Apart from the negative controls, positive fluorescence signal could be detected in sample #1, 4, 8& 22. The other samples showed week signal, which correspond to the raw observations of lateral flow strips. Based on these results, our method could resist the influence from different medium, demonstrating its high feasibility in detecting human samples. Taken together, these results demonstrated that Cas12a-RCFL could be used for the detection of *P. aeruginosa* in clinical samples.

## Discussion

*P. aeruginosa* is a common pathogenic bacterium, which seriously threaten human health^12,13^. In 2017, the World Health Organization (WHO) added *P. aeruginosa* to the short list of antibiotic-resistant pathogens, the so-called ESKAPE group of pathogens. Traditional pathogenic microbial detection methods require the isolation, culture, and biochemical identification of pathogenic bacteria, with the detection cycle ranging from 3 to 5 days. The conventional culture-dependent colony counting method, referred as the gold standard for the detection of microorganisms, is a commonly used method for the detection of *P. aeruginosa* in clinical or environmental samples^14^.Therefore, rapid, sensitive, and specific method for diagnosis of *P. aeruginosa* is paramount in the clinical setting.

At present, a series of rapid identification technologies for bacteria have been developed and optimized using molecular biology and biotechnology, such as qPCR, gene chip technology, phage technology, etc.^6,15–18^. Genome sequencing has limited potential as a predictor of chronic infection and of the adaptive state during infection^19^. Real-time PCR based on PCR technology has been used for early diagnosis of *P. aeruginosa* infection and strain identification^15^. However, due to its requirement for a sophisticated thermocycling instruments, the application in lower source settings was limited, and is difficult to meet the needs of on-site testing in non-laboratory environments^6,7^. Aptamers and immunological techniques were usually applied to develop rapid detection method for *P. aeruginosa*. These assays involve in the use of antibodies. They are often sensitive to the temperature and pH changes, which may lead to the inaccurate test results with false negatives/positives (Zheng et al., 2020, Khatami et al., 2022). Although the methods based on aptamers and immunological techniques commonly exhibit great selectivity, they still suffer from the low sensitivity. All these limits their clinical applications.

The nucleic acid detection platform developed based on the activity of "lateral branch cleavage" of Cas12, cas13 and cas14 has shown great potential in microbial diagnosis^20–22^. Based on Cas12, the development of DETECTER has been promoted, which is highly sensitive, quickly detects individual DNA or RNA, and allows the identification of individual base mismatches. The "collateral cleavage" activity of CRISPR-Cas12a has been used for the detection and control of antibiotic-resistant infections in clinical samples and to identify strains and subspecies of the bacterium^23^. Similarly, upon activation, Cas13a shows non-sequence specific RNAase activity. Shen et al. developed an APC CAS (allosteric probe-initiated catalysis and CRISPR-Cas13a) detection technology without nucleic acid extraction, which can be used for high-sensitivity detection of Salmonella in milk, and the detection sensitivity reached 10 CFU/mL ^24^. Most of the above detection strategies need fluorescence reader which might be an obstacle for the point-of-care testing (POCT).

This study, taking a specific gene of *P. aeruginosa* as target, using amplification reaction tubes and test strips, successfully established an RPA-based CRISPR/Cas12a detection platform for *P. aeruginosa*. Moreover, it can detect rapidly, sensitively, specifically, and portably. The Cas12a-RCFL has been verified by clinical sample and the procedure takes only 45 min (10 + 20 + 15) from DNA extraction to results for naked-eye observation. Compared with other methods, the main superiorities of Cas12a-RCFL are summarized as follows: (1) it takes a short time (45 min) to complete the interpretation of the results; (2) it does not rely on large instruments, it is directly interpreted with naked-eye through the test strip, and if necessary, irradiation from UV light is required; (3) it has high sensitivity; (4) the clinical wound swab sample is verified, making the point-of-care test (POCT) possible in the field.

The isolates of *P. aeruginosa* were collected from the wound samples of 22 patients. The specificity for Cas12a-RCFL was consistent with that of PCR method. However, further investigations are required to gain a better understanding of the clinical feasibility by larger collection of various parts of the body.

By integrating RPA of target DNA to the CRISPR/Cas12a system, we developed a Cas12a-RCFL method, which achieved LOD of 50 CFU/mL with high specificity both in pure and complex samples. This detection method is simple, portable, rapid, and inexpensive, which is applicable for screening of *P. aeruginosa* infection in the clinic.

### Supplementary Information

Supplementary Information.

## Data Availability

The datasets used and/or analyzed during the current study are availablefrom the corresponding author on reasonable request.
