# Supplementary material for: Rapid and sensitive detection of Pseudomonas aeruginosa by isothermal amplification combined with Cas12a-mediated detection
Source: Sci Rep. 2023 Nov 6;13:19199. doi: 10.1038/s41598-023-45766-0 (PMC10628258; doi:10.1038/s41598-023-45766-0)
Supplement: Supplementary file 1 — Supplementary Information. [file 41598_2023_45766_MOESM1_ESM.docx]

**Supplementary Materials**

**
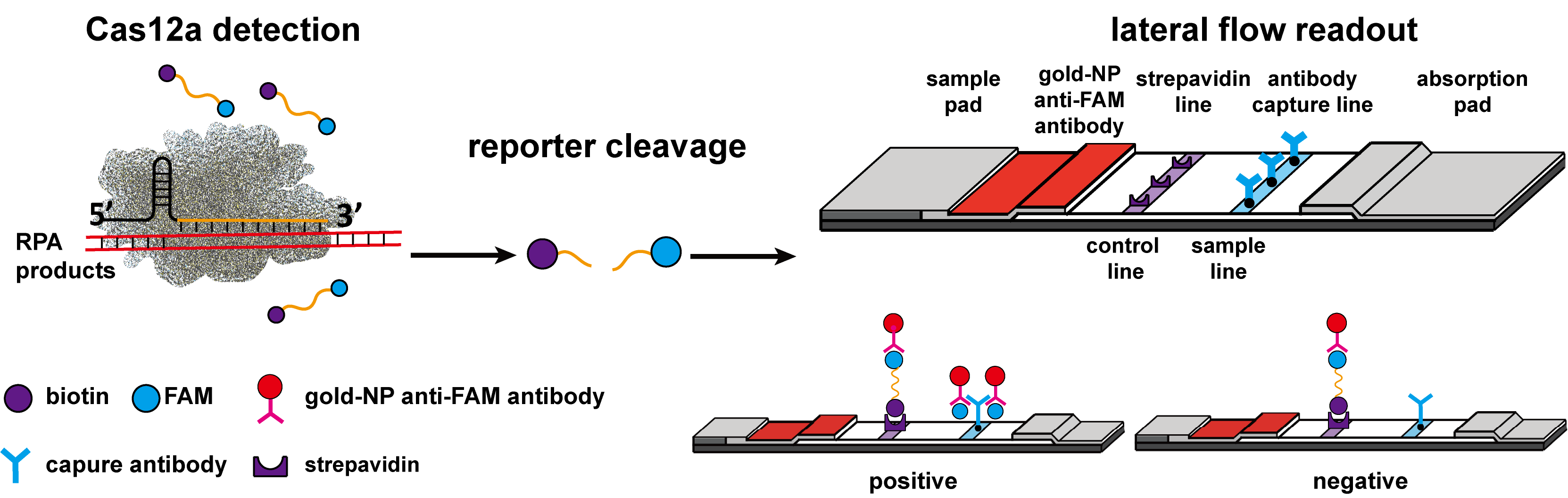
**

**Figure R1 Illumination of CRISPR-based lateral flow readout for detection.**

**Table S1 Sequences of oligonucleotides used in Cas12a-RCF/L protocols.**

|  | Sequences (5' - 3') |
| --- | --- |
| RPA-Forward | CAATATGGAGACCGTTTTCAGGTATTTCAAG |
| RPA-Reverse | TGACTTGCATGCAGGATTACCGTCTATTAGAGC |
| RPA product sequence | CAATATGGAGACCGTTTTCAGGTATTTCAAGGCACGCGGCAGATTGTCACC  TGCTAGGCTGTCCATCCAGTGTAGATGGCTCCAGCTTCGCAGCCAGGTGAA  CTGGCGCTTGGCCAACTGGCGGGTAGCGATGATGCCGCGTTCGGTCATTTC  AGCGTAGGACAGCTTGCCATCCAGGTAATCCCACACCTGACGGTAACCCAC  GGCTCTAATAGACGGTAATCCTGCATGCAAGTCA |
| qPCR- Forward | AACCTGGGAACTGCATCCAA |
| qPCR-Reverse | CTCAGTGTCAGTATCAGTCC |
| PCR- Forward | CAATATGGAGACCGTTTTCAGGTATTTCAAG |
| PCR-Reverse | TGACTTGCATGCAGGATTACCGTCTATTAGAGC |
| crRNA-Pa | UAAUUUCUACUAAGUGUAGAUAGCGUAGGACAGCUUGCCAU |
| ssDNA-FQ | HEX-TATTATT-BHQ1 |
| ssDNA-biotin | 6-FAM-TTTTTTTATTTTTTT-biotin |

**Table S2 Bacterial strains used in this study.**

| Bacteria species | Strain |
| --- | --- |
| *Acinetobacter baumannii* | ATCC 19606 |
| *Klebsiella pneumoniae* | clinical strain identified by our lab |
| *Enterococcus faecium* | ATCC 19433 |
| *Staphylococcus aureus* | ATCC 25923 |
| *Pseudomonas aeruginosa* | PAO1 |
